# Emerging biomarkers in ischemic stroke

**DOI:** 10.20517/2574-1209.2025.58

**Published:** 2025-08-14

**Authors:** Jessie Lee, Peter S. Giannaris, Cigdem Erkuran Yilmaz, Gokhan Yilmaz

**Affiliations:** 1 Molecular, Cellular and Biomedical Sciences, CUNY School of Medicine, New York, NY 10019, USA.; 2 Medical Education, CUNY School of Medicine, New York, NY 10019, USA.

**Keywords:** Ischemic stroke, cerebral ischemia, biomarkers, omics

## Abstract

Ischemic stroke is a devastating global public health problem and the leading cause of acute death and chronic disability. Despite being the diagnostic cornerstone, limitations in neuroimaging, including availability, cost, and therapeutic window, have rekindled interest in biomarker-based approaches. Biomarkers will be employed to facilitate the eventual prediction, early diagnosis, and prognosis of strokes, as well as to inform person-centered medicine. This review summarizes recent advances in the search for biomarkers related to inflammatory, endothelial, metabolic, and neuroaxonal pathways. Interleukin-6 (IL-6), asymmetric dimethylarginine (ADMA), endothelial microparticles (EMP), and homocysteine serve as predictive biomarkers corresponding to vascular risk and inflammatory priming. Glial fibrillary acidic protein (GFAP), D-dimer, and neuron-specific enolase (NSE) are diagnostic markers that can already subtype stroke and estimate lesion burden. Prognostic biomarkers, such as serum neurofilament light chain (sNfL), N-terminal pro-B-type natriuretic peptide (NT-pro-BNP), and growth differentiation factor 15 (GDF-15), are associated with infarct size and long-term outcomes. The -omic sciences (genomic, proteomic, and metabolomic) have discovered defined molecular signatures and panels with high specificity to describe heterogeneity in stroke. Cerebrospinal fluid (CSF) biomarkers and newer imaging modalities, such as those provided through positron emission tomography/computed tomography (PET/CT), offer valuable adjuncts to blood biomarkers in the diagnosis of conditions. Translational potential is hindered by heterogeneity in the transcriptional landscape.

## Graphical Abstract

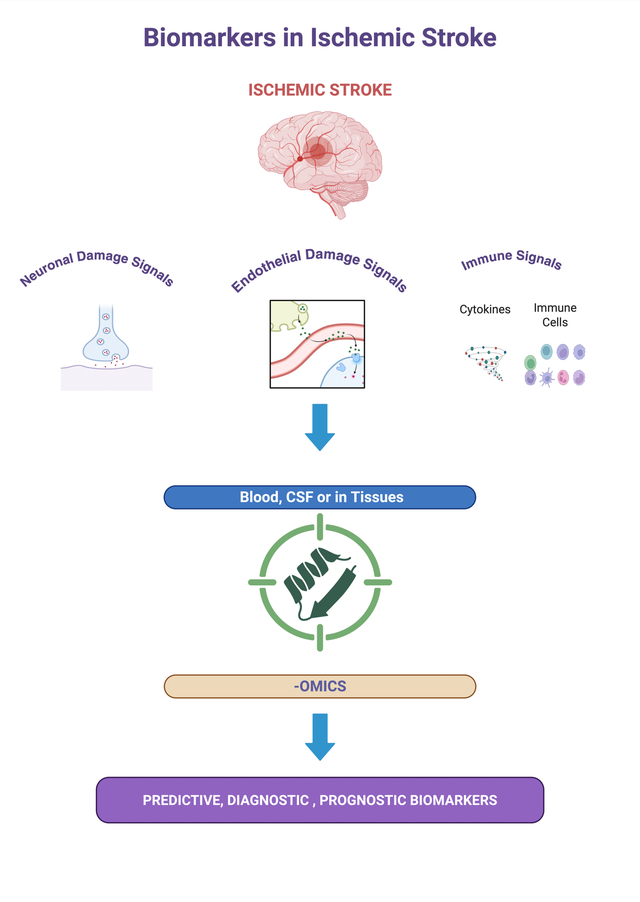

## INTRODUCTION

Ischemic stroke is a devastating disease and the fifth leading cause of death in the United States. It is also one of the leading causes of long-term disability, with an estimated 7.6 million annual stroke survivors. Stroke-related disability places a significant burden on society^[1]^. For example, the estimated cost of stroke treatment and related morbidity costs annually was USD 52.8 billion^[2]^. It also remains a substantial cause of global mortality and chronic disability^[3]^. Multiple pathological pathways are involved in the development of ischemia. These involve the abrupt interruption of cerebral blood flow, resulting in subsequent energy failure due to ischemia, ischemia-induced excitotoxicity, oxidative stress, the initiation of inflammation, and the breakdown of the blood-brain barrier (BBB)^[4]^. Since neurons are the most susceptible tissue to ischemia-associated damage, rapid identification of cerebral ischemic events and prompt intervention decisions are critical in limiting the overall damage associated with cerebral ischemia. Current diagnostic approaches include neuroimaging techniques such as computed tomography^[5]^ (CT) and magnetic resonance imaging (MRI), which are limited by accessibility, feasibility, and cost. Additionally, their use is limited, as they are primarily used for diagnostic purposes.

Stroke pathogenesis is a complex process that encompasses heterogeneous underlying mechanisms stemming from modifiable risk factors and hereditary composition^[6]^. Stroke can be classified into two main categories: ischemic stroke and hemorrhagic stroke. Ischemic stroke, which accounts for the majority of cases, occurs due to the obstruction of cerebral blood flow. Ischemic stroke is further categorized based on etiology into subtypes such as large artery atherosclerosis, small vessel disease (lacunar stroke), cardioembolic stroke, strokes of other determined causes, and cryptogenic strokes (including embolic stroke of undetermined source). Additionally, watershed strokes represent a specific ischemic pattern that occurs in the border zones between major cerebral arteries. Hemorrhagic strokes occur due to bleeding within the brain. Hemorrhagic strokes include intracerebral hemorrhage (ICH) and subarachnoid hemorrhage (SAH), often with an underlying cause, such as hypertension or aneurysm rupture^[7]^. This classification is crucial for guiding clinical management, prognosis, and the development of targeted therapeutic strategies, as well as for validating biomarkers. The use of biomarkers in clinical and experimental settings may provide accessible, rapid, and specific tools that can aid in predicting, diagnosing, and guiding the management of ischemic stroke.

Identification of biomarkers for ischemic stroke has been challenging owing to the heterogeneous nature of ischemic stroke and complex patient profiles related to ischemic diseases. Ischemic stroke is, on the one hand, a vascular event that leads to abrupt ischemia; however, on the other hand, it involves multiple complex pathways of inflammation and tissue repair. In addition, a typical stroke patient would have confounding pathologies such as diabetes, hypertension, coagulation problems, and cardiac rhythm problems, all of which can confound biomarker discovery. Furthermore, this patient group uses multiple pharmaceuticals that target multiple pathways, which further complicates the search for biomarkers. For example, traditional markers of generalized inflammation and vascular events, such as D-dimer and C-reactive protein (CRP), have offered limited specificity in diagnosis and treatment^[8]^. Although both are related to ischemic stroke, CRP and D-dimer lack the precision to distinguish between stroke subtypes or provide insight into the pathogenesis of ischemic stroke. D-dimer and CRP are both nonspecific markers, indicating an ongoing excess of fibrinolysis and general inflammation, respectively. When these markers are used for diagnostic evaluation, limitations such as nonspecific increases in levels due to other non-ischemic pathologies (cancer, inflammatory diseases), timing, and general fluctuations in the levels of these markers should be considered. D-dimer is a general indicator of fibrinolysis and thrombotic events in the body. It can serve as both a marker of a cardioembolic stroke and an ongoing distant thrombotic event. In contrast, CRP is a general inflammatory marker that reflects systemic inflammatory activity; however, it does not offer stroke-type specificity^[9]^.

In this review, we have classified the biomarkers associated with ischemic stroke into three subcategories (predictive, diagnostic, and prognostic). This approach stems from the requirement for clinical applicability and translation of current knowledge in biomarker research. In our approach to categorizing biomarkers by clinically relevant timelines, we aim to establish connections between the current literature and bedside applications. We conducted a full literature review using PubMed using the following search terms: “Ischemic stroke”, “prognostic markers”, “diagnostic markers”, and “predictive markers”. We emphasized selecting clinically relevant studies, peer-reviewed studies in humans, and systematic reviews that were recently published. Additionally, we selected high-quality studies based on omics-based research, including genomics, proteomics, and metabolomics studies, that described new biomarkers with potential translational applications. We focused on studies using large cohorts and multicenter studies of validated biomarker panels for scientific viability and relevance to the current stroke management landscape.

Biomarkers in ischemic stroke can be categorized into predictive, diagnostic, and prognostic subtypes based on their timing and usage in clinical and experimental settings. Predictive biomarkers are measurable indicators of the possibility of developing ischemic stroke. These markers often reflect ongoing vascular inflammation, endothelial dysfunction, or a prothrombotic state associated with increased risk for ischemic stroke. Many candidates in this group are indicators of inflammation, endothelial damage, and prothrombotic state. For example, plasma levels of IL-6^[10]^ and lipoprotein-associated phospholipase A_2_ (Lp-PLA_2_)^[11]^ have been associated with an increased stroke risk in clinical studies^[10,12,13]^.

Although these biomarkers are highly associated with the increased risk of stroke, often clinical history, findings from other laboratory and imaging tests are required to be used jointly for stratifying the risk groups. Diagnostic biomarkers help to identify and recognize the types of cerebral ischemic events during an acute event. For example, GFAP and D-dimer can be used to differentiate between hemorrhagic and ischemic strokes or to indicate a cardioembolic source, respectively^[14,15]^. Diagnostic biomarkers also involve imaging modalities, which have been the gold standard of stroke diagnosis. These include DW MRI and CT perfusion, which enable the rapid identification of the infarct core and penumbra. Tools like the DW Imaging-Perfusion-Weighted Imaging (PWI) mismatch and ASPECTS score are validated, and these can predict stroke severity and subsequent therapeutic response^[5,16,17]^.

A future objective of biomarker studies on ischemic stroke must also be to differentiate between the stroke subtypes, such as lacunar and non-lacunar infarcts. Occlusion of smaller vessels manifests as lacunar strokes with different pathophysiology, as well as clinical characteristics and outcomes from other types of stroke. A large body of data suggest that these differences are reflected in the profiles of biomarkers. Indeed, the study conducted by Jickling *et al.* revealed a 41-gene expression signature that accurately discriminates between lacunar and non-lacunar strokes, further providing evidence of distinct immune response signatures in these two categories^[18]^. There is also a difference in inflammatory and cardiac biomarkers: high-sensitivity C-reactive protein (hs-CRP) is much lower in lacunar than in non-lacunar stroke^[19]^, and serum pro-BNP is much lower in lacunar stroke patients, which demonstrates that they have little cardiac involvement in their pathogenesis^[20]^. Moreover, clinical observations suggest that even a diagnosis based entirely on lacunar syndromes may be subject to error, with a high possibility (up to 16%); thus, there is a need to support the diagnosis with the use of imaging and molecular confirmation^[21]^. Comprehensively, these data support the necessity of subtype-oriented validation of stroke biomarkers to enhance targeted diagnosis and therapy.

Prognostic biomarkers aim to provide information about disease progression, potential complications, and long-term functional outcomes of the patients. For example, sNfL, NT-proBNP, and GDF-15 have shown strong correlations with infarct volume, neurological functional outcomes, and mortality risk in multicenter trials^[22]^. sNfL levels exhibit three distinct phases: an acute increase from 0 to 7 days, followed by a peak from 14 to 21 days. SNfL levels can be used as a diagnostic biomarker to distinguish between acute ischemic stroke and transient ischemic attack^[23]^.

Omics technologies, including genomics, proteomics, and metabolomics, have ushered in a new era in biomarker discovery for ischemic stroke. These applications are capable of capturing complex yet distinct molecular signaling pathways, thereby offering improved specificity and potentially enabling early detection of diseases. Genomics is the study of DNA, focusing on the sequencing, structure, and function of genes. Human genome sequencing provided the basis for our understanding of genetic contributors to stroke risk and response^[24]^. Based on genomics research, NfL, encoded by the NEFL gene, has emerged as a blood-based biomarker of neuronal and axonal damage following stroke. Elevated serum NfL levels are found to be associated with clinical stroke severity at onset and radiographic infarct volumes and future outcomes in stroke patients^[23]^. Proteomics is the culmination of techniques that enable the large-scale analysis of proteins, including their structure, function, and interactions with one another. Mass spectrometry-based proteomics enables the profiling of proteins and has become a central tool for discovering protein biomarkers^[25]^. For example, mass spectrometry-based proteomic characterization in blood clots was used to identify potential biomarkers of acute ischemia. Biomarkers that are isolated from blot clots appear to be correlated with the etiology of the strokes. Pleckstrin is dominant in cardioembolic pathologies, CD59 glycoprotein is dominant in extensive atherosclerosis of the arteries, large artery sclerosis (LAA), and Immunoglobulin Heavy Constant Gamma 1 is dominant in cancer-related thrombi^[26,27]^. In addition, the detection of urinary peptides via proteomic classifiers is proposed to distinguish between ischemic stroke and transient ischemic attack (TIA) in patients with cerebrovascular disease^[28]^. Metabolomics examines the ongoing metabolic signatures of tissue processes, focusing on small-molecule metabolites in cells, tissues, or fluids. This approach provides a dynamic snapshot of the organism’s physiological status^[29]^. Metabolomics-based research has been utilized in the analysis of thrombi associated with stroke, using serum samples from stroke patients and control subjects. Metabolites such as glutamate, serotonin, phospholipids, and triacylglycerols were altered in stroke patients compared to controls^[30]^.

The biomarker development field is promising, as the use of conventional and omics-based biomarker research provides a robust framework. These newly developed biomarkers would allow personalized treatment and prognostic approaches for the patients. Although numerous discoveries have been made in the field, as the number of biomarkers increases, the interpretation of this massive amount of data poses a challenge and requires large-scale analytical methods that potentially utilize artificial intelligence-based models. In the following sections, we aim to review recent progress in identifying predictive, diagnostic, and prognostic biomarkers for ischemic stroke, as outlined in Table 1.

The following abbreviations and symbols are used in the table. IL-6, TNF-α (Tumor Necrosis Factor-alpha), and hs-CRP (High-sensitivity C-reactive Protein) are markers of systemic inflammation. MCP-1 and sCD40L are involved in the recruitment of immune cells and the activation of platelets. EMP, ADMA, VCAM-1 (Vascular Cell Adhesion Molecule-1), and ICAM-1 (Intercellular Adhesion Molecule-1) reflect endothelial dysfunction and vascular inflammation. Lp-PLA_2_ indicates lipid-mediated vascular risk. GFAP, NSE, D-dimer, and OPN serve as diagnostic biomarkers reflecting astrocyte damage, neuronal injury, thrombotic activity, and neuroinflammation, respectively. miR-124 (MicroRNA-124), S100B (S100 Calcium-Binding Protein B), and Tau (Tau Protein) are associated with neuronal damage and BBB integrity. NT-proBNP and hs-TnT (High-sensitivity Troponin T) are cardiac-related markers, while GDF-15 and sNfL are indicators of stress and neuroaxonal damage. *Increased D-Dimer level when used with standard diagnostic imaging tools for diagnosis is a potent adjuvant for ischemic stroke diagnosis^[68]^, as well as follow-up prognosis^[67]^.

## PREDICTIVE BIOMARKERS

### Neuroinflammatory predictive markers

Inflammation is a critical process in the initiation of ischemic stroke. Ischemia initiates activation of circulating immune cells and platelets and increases the expression of cell adhesion molecules on the endothelial surface. Ischemia-associated immune cell recruitment occurs in three phases, where an initial recruitment of polymorphonuclear cells is observed, followed by the adhesion of platelets and mononuclear cells. Proinflammatory cytokines, such as TNF-alpha, IL-1, and IL-6, are increased and further exacerbate the inflammatory response following ischemia^[82]^.

Proinflammatory phenotypes in the endothelium render the organism susceptible to further thrombosis^[44]^. Ongoing inflammation, as measured by increased IL-6 levels, is a potential trigger for the destabilization of atherosclerotic plaques and thromboembolism. A large meta-analysis found that higher IL-6 levels were associated with an increased risk of future ischemic stroke, with stroke risk rising progressively in relation to IL-6 concentration^[31]^.

IL-6 also contributes to endothelial damage and increased BBB permeability in vitro. IL-6 blockade with antibodies appears to protect endothelial cells from the harmful effects of IL-6, underscoring the direct damage to endothelial cells caused by IL-6^[31,32]^. In the CANTOS trial, an increase in the levels of proinflammatory cytokine IL-6 predicted future vascular events independent of traditional risk factors, underscoring its role as a predictive biomarker in clinical settings^[10,83,84]^. IL-6 is also implicated in the remote vascular dysfunction associated with ischemic stroke^[83]^.

High-sensitivity CRP (hs-CRP), although nonspecific, continues to be a reliable predictor of stroke risk and recurrence. Elevated hsCRP levels were associated with an increased risk of total stroke, independent of traditional risk factors^[9]^. In addition, in atrial fibrillation patients, increased CRP levels were correlated to increased stroke risk, secondary vascular events, and mortality^[35]^.

Studies also highlight the predictive use of MCP-1 and soluble CD40 ligand (sCD40L) as biomarkers in ischemic stroke. Measurements of sCD40L in patients who are enrolled in the Clopidogrel in High-Risk Patients with Acute Nondisabling Cerebrovascular Events (CHANCE) trial showed that higher levels of sCD40L increased the risk of recurrent strokes in comparison to the lower levels of sCD40L in patients. Based on this study, an increase in sCD40L levels can predict future stroke incidents in patients with minor strokes and transient ischemic attacks^[38]^. However, in another clinical study, sCD40L levels were found to be negatively associated with stroke risk^[35]^.

MCP-1 is a major player in ischemia-related inflammation, facilitating monocyte recruitment and sCD40L platelet activation, both key events in ischemic stroke^[36,38,82]^. In a prospective significant population-based cohort study, the association between baseline circulating MCP-1 levels and risk of different types of stroke, such as ischemic stroke and hemorrhagic stroke, was evaluated for 16.3 years. A higher chance of stroke was observed in individuals with MCP-1 levels above baseline. Although high MCP-1 levels predicted future stroke incidence, they could not distinguish between hemorrhagic or ischemic types of stroke^[36]^.

MCP-1 has been shown to predict adverse outcomes independently. In a prospective cohort from the Third China National Stroke Registry study (*n* = 10,700), elevated MCP-1 levels were associated with a 35% increased risk of all-cause mortality and a 19% higher likelihood of poor functional outcomes after ischemic stroke or transient ischemic attack. MCP-1-mediated risk increase appears to be mediated by sensitive C-reactive protein, IL-6, and YKL-40 (chitinase-3-like protein 1)^[37]^.

Similarly, sCD40L, a marker of platelet activation and vascular inflammation, has been shown to have prognostic significance in patients with atrial fibrillation. High sCD40L levels showed a 4.6-fold increased risk of future vascular events, including ischemic stroke^[35]^. Although these biomarkers seem to predict ischemic stroke, their use is limited to experimental or clinical studies and has not yet been translated to clinical use.

Experimental studies have shown that T lymphocytes and proinflammatory cytokine interferon-γ are key mediators of ischemic brain injury and infarct progression^[85]^. Neutrophil-to-lymphocyte ratio (NLR) has been proposed as a predictive biomarker for ischemic stroke events. In a prospective cohort of healthy adults, elevated NLR predicted ischemic stroke incidents and improved risk stratification^[86]^. In support of these findings, in a 2024 cohort study in a rural, low-income population, higher NLR levels were found to be associated with increased stroke incidence^[87]^. In a large group of patients with atrial fibrillation, an NLR ≥ 3 was associated with a 1.4-fold higher risk of stroke^[88]^.

### Endothelial dysfunction predictive markers

Endothelial dysfunction, characterized by impaired vasodilation, increased vascular permeability, and prothrombotic phenotype, is a well-established precursor of cerebrovascular events. Among the new biomarkers of this dysfunction are circulating EMP- submicron vesicles released from endothelial cells following activation or apoptosis. Circulating EMP have also been recognized as biomarkers for endothelial injury; their increase reflects active endothelial apoptosis and shedding^[40]^. Mechanistically, EMP propagate vascular inflammation by carrying proinflammatory and pro-coagulant cargo to distant vascular beds^[89]^. Elevated levels of EMP signify active endothelial injury and correlate with systemic vascular damage^[90,91]^. Mechanistically, EMP cause vascular inflammation by delivering proinflammatory and pro-coagulant cargo, including ICAM-1, tissue factor, and thromboxane A2, which are accountable for leukocyte adhesion and thrombotic cascades in remote vascular beds^[92]^. EMP can also inhibit angiogenesis by downregulating the expression of VEGF and eNOS, thereby exacerbating endothelial dysfunction^[89]^. Clinically, elevated EMP levels are observed in patients with acute ischemic stroke as well as in those with other atherothrombotic illnesses. Interestingly, phospholipid-rich EMP levels were found to be higher in patients with acute stroke, and EMP carrying ICAM-1 have been found to correlate with stroke lesion volume and functional outcome, underlining their potential role as prognostic biomarkers in ischemic stroke management^[39]^.

ADMA is an endogenous metabolite and can inhibit nitric oxide synthase. ADMA induces vascular dysfunction. ADMA inhibits nitric oxide (NO) production in the vessel, thereby impairing endothelial-derived vasodilation and promoting platelet aggregation. Elevated plasma ADMA levels also potentially contribute to atherosclerosis by increasing the carotid intima-media thickness ratio^[42]^. Increased ADMA levels are proposed as predictive risk factors in ischemic stroke. Individuals who have increased ADMA levels have been reported to have a stroke risk ratio of 1.60^[43]^. These increased levels can also be predictive of future stroke events, particularly in individuals without apparent vascular diseases^[42]^. ADMA is also considered a therapeutic target in ischemic events. Although arginine supplementation to reduce ADMA has been proposed as a therapeutic intervention for endothelial dysfunction, it has not been widely adopted in everyday clinical use^[41]^.

Following an ischemic insult, cerebral endothelial cells increase their expression of cell surface adhesion molecules that mediate the recruitment of leukocytes and platelets. These molecules serve as attachment sites for the recruitment of leukocytes and platelets. Expression of cell adhesion molecules contributes to the disruption of the BBB and the development of infarcts^[44,82]^. An increase in adhesion molecules such as VCAM-1 and ICAM-1 is indicative of endothelial activation. Higher expression of these molecules is associated with increased risk of thrombus formation in the cerebral vasculature^[93,94]^. Elevated baseline serum levels of soluble intercellular adhesion molecule-1 (sICAM-1), an inflammatory marker, were independently associated with a more than twofold increased risk of future ischemic stroke in patients with chronic coronary heart disease. This association was robust for large, disabling cardioembolic strokes, highlighting sICAM-1’s potential as a predictive biomarker^[47]^.

### Metabolic and coagulation pathways-associated predictive markers

Disruption of ATP-providing metabolic pathways and activation of coagulation cascades are integral to stroke pathogenesis. Elevated Lp-PLA_2_ catalyzes the hydrolysis of oxidized phospholipids in LDL, generating proinflammatory and proatherogenic products that enhance plaque vulnerability. Elevated Lp-PLA_2_ levels have been independently associated with increased risk of ischemic stroke, as shown in the ARIC study by Ballantyne *et al.* (2005). Analysis further confirmed that both Lp-PLA_2_ mass and activity are predictive of ischemic stroke, coronary events, and vascular mortality, supporting its role as a clinical biomarker^[11,49]^.

Hyperfibrinogenemia was also associated with an increase in blood viscosity and platelet aggregation, which creates a condition conducive to thrombus formation. Fibrinogen has also been proposed to be a predictor of stroke outcome and complications^[50]^. Hyperfibrinogenemia (fibrinogen level > 3.5 g/L) was independently associated with increased long-term mortality in ischemic stroke patients regardless of age and stroke severity^[51]^. In a 2024 prospective cohort study, a nonlinear association was observed between serum fibrinogen levels and poor 3-month functional outcomes, with values greater than 2.74 g/L indicating a strong prediction of disability risk^[50]^. The levels of fibrinogen were also found to be positively associated with poorer early neurological worsening^[52]^, suggesting its implication in the identification of patients at risk of worsening within a short period of stroke onset^[53]^. Moreover, elevated fibrinogen levels above a critical value were predictive of the development of stroke-associated pneumonia, a common and serious acute ischemic stroke complication^[54]^.

Higher levels of homocysteine have been continuously correlated with heightened risk and adverse outcomes in ischemic stroke. Studies show that even low levels of homocysteine, such as 10 micromol/L, could be a predictor of the outcome following ischemic stroke^[55]^.

A meta-analysis showed that homocysteine was an accurate biomarker to predict ischemic stroke among populations^[56]^. Similarly, elevated plasma homocysteine is associated with early neurological deterioration among acute stroke patients^[57]^, while a correlation between elevated levels and increased risk of recurrent vascular events is shown^[58]^. Whether interventions that target reducing homocysteine would improve outcomes after ischemic stroke remains unclear. These findings confirm homocysteine as a clinically relevant predictive and prognostic biomarker in the management of stroke.

Recent lipidomic studies have also identified specific lipid alterations with a higher risk of stroke. Notably, decreased lysophosphatidylcholine (LPC) levels and elevated ceramide levels have been associated with proinflammatory states and atherogenesis. LPC and ceramide both facilitate endothelial mitochondrial dysfunction and augment the differentiation of immune cells into proinflammatory phenotypes^[95]^, thereby accelerating the atherosclerotic process. Ceramides appear to be predictive of the extent of stroke-induced damage in clinical studies^[59,60]^. In addition, several dysregulated sphingolipids, such as SM 38:1 and Cer 34:1, were correlated with high white matter hyperintensities among chronic cerebral ischemia patients^[96]^, highlighting the potential of lipidomic profiling in stroke risk stratification.

## DIAGNOSTIC BIOMARKERS

### Blood-based diagnostic markers

Rapid and precise stroke diagnosis in ischemic stroke is crucial for early emergency intervention. GFAP is a prominent astrocyte intermediate filament protein that rises quickly in hemorrhagic stroke but has a blunted or delayed response with ischemic stroke. When used in conjunction with ubiquitin C-terminal hydrolase L1, the levels of these two markers enable a distinction between hemorrhagic and ischemic strokes^[62]^. Mechanistically, GFAP release is a marker of astrocyte damage following mechanical injury rather than ischemic necrosis; therefore, its diagnostic specificity is limited in pathologies that include astrocyte damage. Several studies support the application of GFAP as a biomarker in differentiating ischemic stroke (IS) from ICH. In a multicenter, prospective study, it was demonstrated that GFAP serum levels were significantly elevated in ICH patients compared to IS patients, with high sensitivity and specificity at 4.5 h from symptom onset^[97]^. It was also demonstrated that elevated GFAP levels correlated with ICH risk and may distinguish stroke subtypes effectively^[63]^. Subsequently, ultrasensitive immunoassays are utilized to validate GFAP as a rapid diagnostic tool, with increased intensity in ICH patients and delayed increases in IS cases^[61]^. GFAP has dual potential as both a diagnostic and prognostic marker. Increase in soluble GFAP following ischemic stroke has been an independent indicator of clinical outcomes and rehabilitation measured by National Institutes of Health Stroke Scale (NIHSS) following acute ischemic stroke, Trunk Control Test (TCT), Functional Ambulation Classification (FAC), and Functional Independence Measure (FIM) scores 3 months following the acute ischemic stroke^[64]^. In addition, when combined with NIHSS GFAP measurements at 48 h following endovascular embolectomy, GFAP measurements were strongly correlated with infarct volume and 3-month outcomes^[65]^. These findings demonstrate the promise of GFAP for clinical use in guiding early stroke management therapies.

NSE, an enzyme of glycolysis in neurons, increases in the serum following neuronal injury. It serves as a surrogate measure for the extent of neuronal injury, but is compromised by increases in other neurological conditions^[98]^. Increased levels of NSE in serum are correlated with infarct size and neurological severity, making it a useful diagnostic marker in acute ischemic stroke^[99,100]^. Surprisingly, Pandey *et al.* demonstrated a positive correlation between NSE levels and the size of the infarct. Inversely, Glasgow Coma Scale scores imply greater neurological injury with higher levels of NSE^[66]^. These findings validate the use of NSE not just as a marker of diagnosis but also for early risk stratification and prediction of outcome in ischemic stroke patients.

D-dimer, a fibrin degradation product, has also emerged as an effective diagnostic marker for recognizing cardioembolic stroke. The levels of D-dimer have been determined to be substantially elevated in the presence of cardioembolic stroke compared to ischemic strokes, indicating chronic fibrinolysis due to excess clot burden and helping identify etiology on presentation^[15]^. Furthermore, elevated levels of D-dimer correlate with stroke severity and have been shown to forecast future embolic strokes, particularly in patients harboring occult atrial fibrillation or cancer^[67]^. Each of these findings makes the utilization of D-dimer not only for acute diagnosis but also for risk stratification, as well as the management of chronic stroke.

Osteopontin (OPN), an immunomodulatory matrix protein, is emerging as a blood biomarker for ischemic stroke. Elevated plasma levels of OPN have been associated with higher mortality and severe disability at one year following stroke, reflecting the intensity of neuroinflammation and breakdown of the BBB^[69]^. Moreover, thrombin-cleaved N-terminal domains of OPN have been identified as biomarkers of acute atherothrombotic ischemic stroke, with the potential to enable early diagnosis of stroke subtypes if measured high within 3 h from stroke onset^[70]^. It is also of note that stem cells that infiltrate the brain following ischemic stroke exhibit a 101-fold increase in OPN expression compared to naïve stem cells^[101]^.

### Cerebrospinal fluid-based diagnostic markers

CSF examination provides direct evidence of the biochemical environment of the central nervous system following stroke. MicroRNAs (miRNAs), particularly miR-124 and miR-9, were elevated in CSF following ischemic stroke and are vital controllers of gene networks in neuronal survival and apoptosis^[71]^. Increased miR-21 levels were associated with a 6.2-fold increase in stroke, while decreased miR-221 increased stroke risk by 10.4 times^[102]^. In addition, the levels of miR-124–3p, miR-125b-5p, and miR-192–5p correlate with adverse outcomes, suggesting their potential use as diagnostic and prognostic biomarkers in ischemic stroke^[72]^.

S100B, a calcium-binding protein of astrocyte origin, has been investigated as an ischemic stroke biomarker. Elevated CSF and serum levels of S100B have been associated with blood-BBB disruption, infarct volume, and neurological severity. For example, serum levels of S100B are predictive of a malignant infarct course of acute middle cerebral artery stroke^[74]^. Additionally, S100B acts on endothelial cells, enhancing BBB permeability and promoting secondary brain injury through Receptor for Advanced Glycation End products (RAGE), thereby activating inflammatory pathways. S100B- RAGE axis has also been found to promote BBB disruption by activating inflammatory signaling pathways in cerebral ischemia. SB100/RAGE levels appear to differentiate between ischemic and hemorrhagic cerebrovascular events^[103]^. High S100B and low soluble RAGE levels were detected in the blood of hemorrhagic stroke patients compared with those presenting with ischemic stroke^[73]^. In addition, elevated S100B levels have been linked with poor functional outcomes following stroke^[73,74]^. The findings demonstrate the importance of S100B as a prognostic and diagnostic biomarker in ischemic stroke, suggesting underlying neuroinflammatory processes and BBB disruption.

Tau protein is an axonal microtubule-associated protein that plays a central role in axonal microtubule stabilization in neurons^[104]^. In acute neuronal injury, such as in ischemic stroke, tau is released into CSF after axonal degeneration. Tau protein was found to be detectable within 6 h after stroke, with a peak after 3–5 days, and levels were correlated with infarct volume and functional disability 3 months after the onset of stroke. Elevated CSF tau levels are associated with infarct size, neurological deterioration, and poor functional outcome^[75,76]^. These findings validate the use of tau as a prognostic and diagnostic marker of the extent of neuronal damage in ischemic stroke.

### Imaging-based diagnostic markers

Advanced neuroimaging techniques remain the gold standard for stroke diagnosis. Among these, DWI MRI is significant because it can detect cytotoxic edema within minutes after ischemia occurs, with high sensitivity to acute infarction. DWI quantifies restricted water molecule diffusion, which is apparent once cells become energy-depleted and ionic gradients fail, leading to cellular swelling^[105]^. The DWI hyperintensity that ensues accurately delineates infarcted brain and is predictive of lesion expansion, especially in patients with reduced collateral circulation^[106,107]^. Such qualities make DWI not only a diagnostic cornerstone but also a prognostic tool for patient stratification and therapy planning. PWI is often used in conjunction with DWI. The use of both imaging modalities enables the visualization of the ischemic penumbra, which can potentially be salvaged. DWI-PWI mismatch is a crucial imaging biomarker to consider when evaluating eligibility for thrombolysis and endovascular thrombectomy^[108]^.

Susceptibility-weighted imaging (SWI) MRI enhances the detection of cerebral microbleeds and hemorrhagic transformation, distinguishing between primary ICH and ischemic stroke with hemorrhagic transformation^[109]^. Outside of MRI environments, CT perfusion (PCT) imaging provides rapid approximations of cerebral blood flow (CBF), cerebral blood volume (CBV), and mean transit time (MTT) as an effective surrogate for penumbral imaging^[110]^.

## PROGNOSTIC BIOMARKERS

### Neuroinflammatory prognostic markers

Inflammatory cytokines play an important role in long-term outcomes after ischemic stroke. Elevated plasma IL-6 levels within the first 24 to 72 h of ischemic stroke are significantly associated with larger infarct volumes, greater stroke severity, and unfavorable outcomes at 90 days^[111,112]^. IL-6 contributes to vascular inflammation by promoting the expression of adhesion molecules and MMPs, leading to BBB breakdown and further neuronal damage in ischemic stroke^[10]^.

Another proinflammatory cytokine, TNF-α, has been implicated in poor outcomes following ischemic stroke. Increased TNF-α levels have been associated with larger infarct sizes and heightened neuroinflammation. TNF-α acts through the typical proinflammatory NF-κB signaling cascade to exert its harmful effects in ischemia. Elevated TNF-α may also contribute to the increased risk of post-stroke infections and delayed recovery^[33,34]^. CRP, measurement of NLR, and interleukin-10 (IL-10) are used as predictors of functional outcomes after stroke. These markers may be used together to improve risk stratification and facilitate more individualized post-stroke treatment^[113]^.

Neutrophil-to-lymphocyte ratio has also been regarded as an indicator of systemic inflammation, and higher NLR in patients was independently associated with greater stroke severity on presentation, worse 3-month functional outcome, and greater short-term mortality^[114]^. Similarly, the lymphocyte-to-monocyte ratio (LMR) has emerged as yet another available and reliable biomarker. LMR levels were significantly decreased with more serious strokes and with poor functional recovery at 90 days^[115]^. T-lymphocyte subpopulation alterations, particularly the CD4^+^ to CD8^+^ ratio, are being explored for their immunomodulatory impacts on post-stroke immune responses. Alterations in these subsets have been associated with complications of stroke and may be indicators of immune system dysregulation after the acute and subacute period of ischemia^[116,117]^.

### Vascular prognostic markers

NT-proBNP is secreted from stretched myocardium. It is often increased in cardioembolic stroke patients compared to patients with other stroke subtypes^[22]^. Elevated levels of NT-proBNP are independently associated with poor functional outcome and elevated 90-day mortality. Recent clinical research has demonstrated that NT-proBNP levels above a critical threshold of 476 pg/mL predict a modified Rankin Scale score of 3–6, indicating severe disability or death following ischemic stroke^[77]^. NT-proBNP is a marker of underlying cardiac dysfunction, such as atrial fibrillation and heart failure, which can impair cerebral perfusion and recovery. Individuals with NT-proBNP levels exceeding 82.2 pg/mL have a twofold higher risk of stroke compared to those with levels below 20.4 pg/mL^[78]^. These findings underline the use of elevated NT-proBNP levels as a prognostic biomarker for stroke risk stratification.

Cardiac hs-TnT, which is a myocardial injury biomarker, tends to be commonly elevated after ischemic stroke and is related to autonomic dysregulation as well as to in-hospital complications, particularly in patients with insular cortex infarction. Elevated hs-TnT has also been revealed to relate to increased risk for recurrent vascular events and death and has proved itself to be helpful as a prognostic marker^[79]^.

GDF-15, a cytokine induced by mechanical and oxidative stress, has been reported to be capable of predicting recurrent vascular events and all-cause mortality after ischemic and hemorrhagic stroke. Patients with higher GDF-15 levels had a higher risk of future stroke and all-cause death. Mechanistically, GDF-15 is known to modulate inflammatory signaling and has been implicated in preventing post-stroke neural repair by inhibiting cellular proliferation^[80]^.

Elevated levels of soluble VCAM-1 and ICAM have been implicated in cerebral ischemic events and found to be associated with poor prognosis following ischemic stroke^[45,46]^. In addition, in humans, genetically elevated higher soluble ICAM-1 and E-selectin levels were found to be associated with poor prognosis following ischemic stroke^[48]^.

Finally, peripheral vascular dysfunction has been described in animal models following ischemic stroke, suggesting that prolonged microvascular dysfunction may play a role in blood pressure liability and impaired systemic recovery. These alterations may provide translational data into vascular biomarkers predictive of post-stroke hemodynamic complications. Yilmaz *et al.* (2024) demonstrated that compromised peripheral vascular function persists following ischemic stroke in mice, suggesting that modifications in microvascular reactivity may contribute to post-stroke blood pressure instability and impaired systemic recovery^[83]^. These findings may have translational relevance to determine vascular biomarkers predictive of hemodynamic complications following stroke in humans.

### Neuronal injury prognostic markers

Markers of neuroaxonal injury offer key insights into the extent of irreversible brain damage following ischemic stroke. sNfL, a structural protein released after axonal damage, has been recognized as a predictive biomarker after stroke^[22,23,81,118]^. Tau protein, found in both CSF and serum, reflects the magnitude of neuronal loss; elevated tau levels have been associated with poor functional recovery and persistent cognitive deficits following a stroke^[75]^.

S100B, a calcium-binding protein secreted by astrocytes, is another marker of neuronal injury. Persistent elevation of S100B levels has been linked to larger infarct size, cerebral edema, and hemorrhagic transformation. Beyond blood biomarkers, neuroimaging tools such as infarct core volume, collateral circulation, and early ischemic changes- quantified by the Alberta Stroke Program Early CT Score (ASPECTS)- continue to serve as key predictors of prognosis and therapeutic windows in the care of acute stroke^[5]^.

## OMICS TECHNOLOGIES AND MULTI-BIOMARKER PANELS

**Figure 1.**
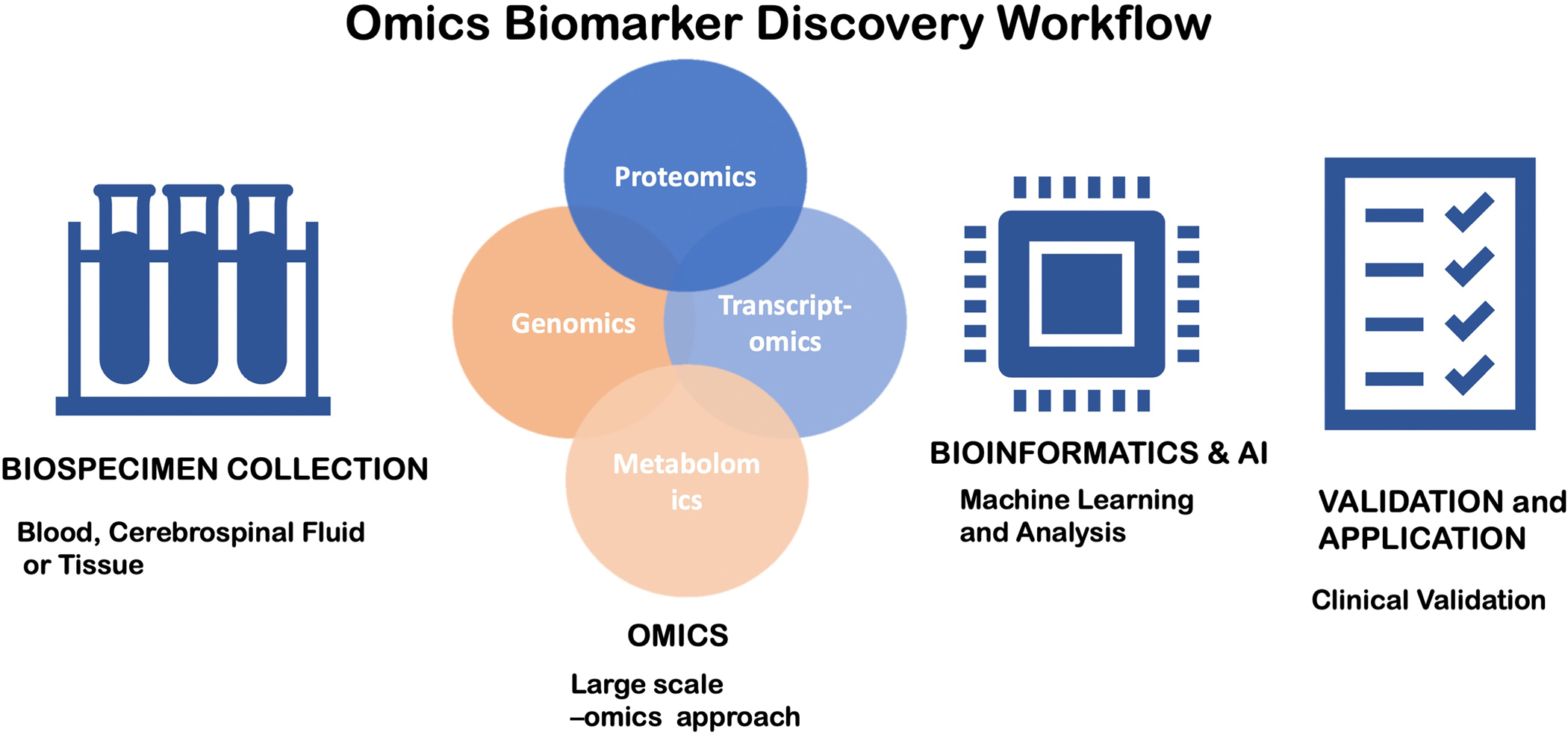
Workflow for Omics Biomarker Discovery. A flowchart illustrates the step-by-step process of omics-based novel biomarker search in ischemic stroke. 1. Biospecimen Collection: Samples are collected from human beings or animals. Time of collection and quality are essential. 2. Omics Analysis: High-throughput processes such as genomics, transcriptomics, proteomics, and metabolomics are employed. Large volumes of gene expression information, protein expression, metabolite changes, and regulatory RNA patterns are generated. 3. Bioinformatics and artificial intelligence: Machine learning, pattern recognition, and network analysis algorithms for target identification and reduction of false positives. 4. Validation and Application: Validation with independent large clinical cohorts.

The integration of omics techniques, such as transcriptomics, proteomics, metabolomics, and epigenomics, has been of great value for biomarker discovery in stroke. These high-throughput platforms can analyze numerous molecules simultaneously, and as a result, the identification of complex biomarker signatures, rather than individual markers, is feasible [Figure 1].

Plasma and CSF proteomic analysis led to the discovery of candidate biomarkers of inflammation, coagulation, and extracellular matrix remodeling pathways. Fibrinogen isoforms, apolipoproteins, and complement factors are shown to increase early after stroke onset, measured by mass spectrometry-based proteomics^[119]^. Large-scale plasma metabolomic profiling has yielded novel biomarkers. The use of mass spectrometry in conjunction with high-throughput proteomics is promising for estimating ischemic stroke onset, particularly within the therapeutic time window for antithrombotic therapies. In a recent study, a two-protein panel consisting of EPB42 and PEBP1 successfully differentiated between early-onset and late-onset ischemic stroke within a 4.5-h time frame^[120]^. In addition, biliverdin and nicotinamide N-oxide are potential markers for the onset of acute ischemic stroke within 4.5 h, thus opening up possibilities for intravenous thrombolysis in patients with an unknown onset time, until large, independent cohorts validate this finding^[121]^.

Transcriptome profiling by RNA sequencing has highlighted the dynamic management of circular RNAs (circRNAs) and long non-coding RNAs (lncRNAs) in response to acute ischemia. circDLGAP4 and circHECTD1, for example, are involved in maintaining BBB integrity and inflammation, and could be targeted as diagnostic biomarkers^[122]^. The use of multi-biomarker panels improves diagnostic accuracy. Panels of inflammatory cytokines (e.g., IL-6, TNF-α), biomarkers of neuronal injury (e.g., NSE, sNfL), and coagulation biomarkers (e.g., D-dimer) have demonstrated greater sensitivity and specificity than individual markers. Machine learning techniques are increasingly being used to enhance panels for personalized risk stratification and therapy selection. For example, a machine learning algorithm using neural networks distinguished survivors from non-survivors in a year based on multiple criteria, including NIHSS, cIMT, age, IL-6, TNF-α, hsCRP, Protein C, Protein S, vWF, and platelet endothelial cell adhesion molecule 1 (PECAM-1)^[123]^.

## LIMITS OF CLINICAL BIOMARKERS

While there is an acceleration in the discovery of novel biomarkers for ischemic stroke, translation of these discoveries to clinical applications faces certain limitations. The heterogeneity of stroke pathology involves various etiologies, different patient populations, comorbidities, and dynamic temporal profiles for individual cases. These factors result in markedly variable biomarker expression across studies and populations. For example, a meta-analysis of 3,494 participants showed that, compared to single-biomarker measurement, multiple blood biomarkers demonstrated diagnostic promise, although accuracy varied in inpatient versus outpatient settings, with reported comorbidities, and among different ethnic backgrounds^[124]^. A second limitation is the preanalytical and analytical variability, including a lack of standardization in sample collection, storage, and processing, as well as the use of different methodologies in various biomarker measurements. These factors undermine the comparability and reproducibility of biomarkers^[125,126]^. Addressing these challenges requires large-scale, standardized, multicenter studies with transparent protocols, so that biomarker-based prognostic and diagnostic applications are robust across diverse populations and clinical contexts.

## CONCLUSION

### Bridging experimental and clinical biomarkers

As we gain greater insight into ischemic stroke pathogenesis and advancements are made in our ability to interrogate large datasets with machine learning, several platform-based biological assays hold promise for the future of biomarker-directed, personalized care of stroke. Among the issues in biomarker studies of stroke, one of long-standing importance is a paradigmatic divide between preclinical discovery and clinical implementation. While animal models provide controlled systems for establishing mechanistically meaningful markers, their varying physiology and limited simulation of human comorbidities generally limit external validity.

The shortage of biomarkers in the clinic is due to the labor-intensive and time-consuming process of discovery and identification. It consists of a discovery phase, a small-scale clinical feasibility phase, a large-scale clinical validation phase, and regulatory approval and clinical implementation phases. While several markers, such as EMP and miRNAs, exhibit robust changes in expression in rodent models of stroke, their clinical validity remains to be established in humans. It requires bridging protocols for sample collection and assay performance, as well as cross-validation of outcomes across various human cohorts, and the acceptance of universal endpoints for biomarker performance, to translate laboratory results into the clinic.

**Figure 2.**
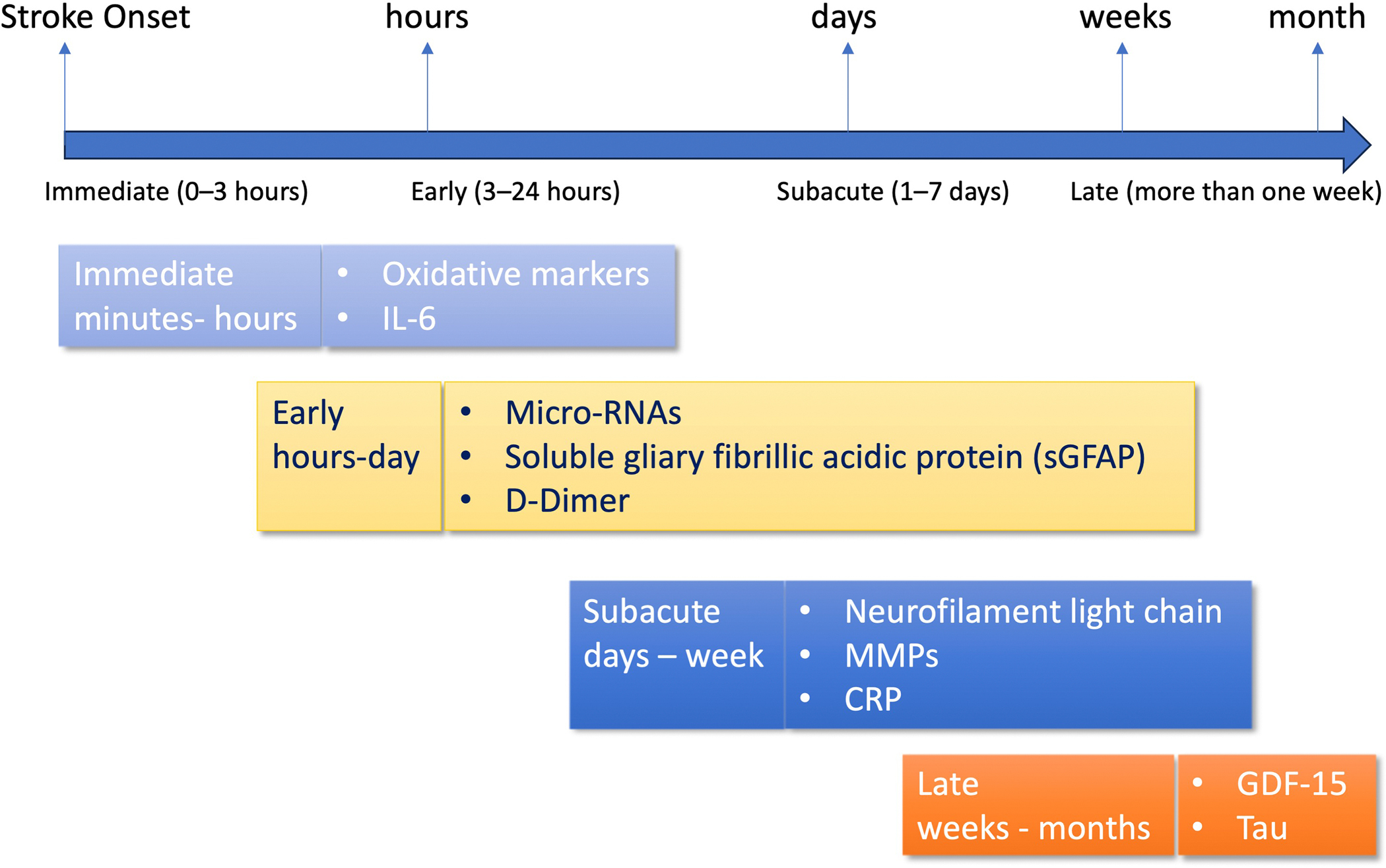
Timing of Biomarker Detection in Ischemic Stroke. The time course of biomarker detection is shown following the onset of ischemic stroke: immediate (0–3 h), early (3–24 h), subacute (1–7 days), and late (more than one week). The rapid appearance of reactive oxygen species and IL-6 characterizes early inflammatory and oxidative stress reactions. In 0–3 h, GFAP, microRNAs (e.g., miR-124), and D-dimer levels increase. This enables subtype differentiation of stroke. Subacute phase biomarkers, such as CRP, sNfL, and MMPs, indicate ongoing neuroinflammation, tissue damage, and axonal injury. During the late phase, chronic increases in GDF-15 and tau protein correlate with chronic damage and long-term prognosis.

Understanding the time course of biomarker release is crucial for their application in clinical practice for stroke treatment. Biomarkers vary in their onset, peak, and duration after stroke onset; therefore, time-resolved analysis is necessary. For example, D-dimer, GFAP, and 8-iso-prostaglandin F2α (8-iso-PGF2α) can be detected within minutes to 3 h of a stroke. IL-6, NSE, S100B, and miR-124 usually increase within 3–12 h of a stroke. Tau protein, NT-proBNP, hs-TnT, and EMP increase within 12–48 h. Late biomarkers that increase over time and are quantifiable include GDF-15, sNfL, ceramide, and acylcarnitine [Figure 2]. By integrating biomarker research and discovery with neuroimaging and history, accurate biomarkers could be used in the clinic. To hasten the transition of biomarkers to the clinic, a stepwise process is required: (1) valid multicenter cohort validation; (2) development of low-cost, quick point-of-care assays; and (3) integration into multimodal diagnostic algorithms with imaging and clinical scales. Validation and Use of Biomarkers in clinical settings will require collaboration among basic scientists, clinicians, bioinformaticians, and regulatory agencies to overcome current challenges. Finally, standardizing protocols for measuring the biomarker and validating them across various stroke subtypes and clinical environments should be a priority for future studies. It is imperative that lacunar and non-lacunar subtypes can be distinguished, as they correlate with specific pathophysiological and biomarker patterns. Future large-scale, prospective, collaborative studies encompassing molecular signatures, imaging, and clinical data will be vital to improving the diagnostic capacity of new, emerging biomarkers and translating them into individualized stroke management policies.

## Figures and Tables

**Table 1.** Key biomarkers in stroke based on their use for predictive, diagnostic, or prognostic purposes

Biomarker	Source	Type	Mechanism/Clinical use	References	Study type

IL-6	Plasma	Predictive	Inflammation, endothelial activation	[10,31,32]	Meta-analysis, Review, Experimental
TNF-α	Plasma	Predictive	Proinflammatory cytokine, NF-*κ*B activation	[33,34]	Review, Experimental
hs-CRP	Plasma	Predictive	Systemic inflammation	[9,35]	Prospective cohort, Observational
MCP-1	Plasma	Predictive	Monocyte recruitment	[36,37]	Meta-analysis, Registry-based prospective study
sCD40L	Plasma	Predictive	Platelet activation	[35,38]	Predictive clinical cohort, Observational
EMP	Plasma	Predictive	Endothelial apoptosis	[39,40]	Clinical study, review
ADMA	Plasma	Predictive	NO inhibition, endothelial dysfunction	[41–43]	Review, Epidemiologic study, Meta-analysis
VCAM-1	Plasma	Prognostic	Leukocyte adhesion	[10,44–46]	Review, Experimental, Observational outcomes
ICAM-1	Plasma	Predictive, Prognostic	Endothelial activation	[39,45–48]	Clinical study, Nested case-control study, Observational clinical study, Review, Clinical genetic epidemiological study
Lp-PLA_2_	Plasma	Predictive	LDL oxidation, plaque instability	[11,49]	ARIC prospective study, Meta-analysis
Fibrinogen	Plasma	Predictive/Prognostic	Hypercoagulability	[50–54]	Prospective cohort studies, Prognostic stroke outcome
Homocysteine	Plasma	Predictive	Endothelial injury	[55–58]	Meta-analysis, Prospective studies
Ceramides	Plasma	Predictive	Lipid metabolism dysregulation	[59,60]	Clinical observational, FINRISK population-based cohort study
GFAP	Plasma	Diagnostic/Prognostic	Astrocyte injury	[61–65]	Prospective cohort, Diagnostic biomarker validation studies
NSE	Plasma	Diagnostic	Neuronal damage	[66]	Clinical correlation study
D-dimer*	Plasma	Diagnostic/Prognostic	Fibrinolysis	[15,67,68]	Prospective cohort, Meta-analysis
OPN	Plasma	Diagnostic	Neuroinflammation	[69,70]	Observational study, Experimental biomarker validation
miR-124	CSF	Diagnostic	Gene regulation post-injury	[71,72]	Prospective, non-interventional clinical study, Prospective cohort study
S100B	CSF/Plasma	Diagnostic	BBB breakdown	[73,74]	Longitudinal prospective study, Clinical validation study
Tau	CSF/Plasma	Diagnostic	Neuronal degeneration	[75,76]	Clinical Prospective, Clinical Observational Study
NT-proBNP	Plasma	Prognostic	Cardiac stretch	[22,77,78]	Prospective NT-cohort, Meta-analysis, and confirmation
hs-TnT	Plasma	Prognostic	Myocardial damage	[79]	Prospective cohort study
GDF-15	Plasma	Prognostic	Stress/inflammation	[80]	Prospective cohort outpatient study
sNfL	Plasma	Prognostic	Axonal injury	[22,23,81]	Meta-analysis, Prospective cohort studies

Biomarkers are classified as predictive (risk indicators before a stroke), diagnostic (used during an acute stroke to determine the type or mechanism), or prognostic (predicting outcomes or complications).

## Data Availability

Not applicable.
